# Imaging of inferior vena cava normal variants, anomalies and pathologies, Part 2: Acquired

**DOI:** 10.4102/sajr.v27i1.2694

**Published:** 2023-12-08

**Authors:** Ranjit K. Chaudhary, Pankaj Nepal, Shruti Kumar, Elina Gupta, Nikita Sangroula, Arpit Nagar, Vijayanadh Ojili

**Affiliations:** 1 Department of Radiology, St. Vincent’s Medical Center, Bridgeport, United States of America; 2 Department of Radiology, Massachusetts General Hospital, Boston, United States of America; 3 Department of Radiology, University of Arkansas, Little Rock, United States of America; 4 Department of Radiology, Ohio State University Wexner Medical Center, Columbus, United States of America; 5 Department of Radiology, University of Texas Health, San Antonio, United States of America

**Keywords:** IVC, vena cava, IVC neoplasms, tumour thrombus, IVC perforation, megacava, slit-like IVC, Budd-Chiari Syndrome, fistula, pseudolesion

## Abstract

The inferior vena cava (IVC) is an uncommon site for primary pathologies and secondary involvement is also infrequent, but involvement of the IVC can often drastically change management. It is therefore important to be cognizant of IVC pathologies. This review discussed common and rare neoplastic and non-neoplastic pathologies of the IVC as well as pathology mimics. Primary and secondary neoplasms can lead to tumour extension or bland thrombus formation and it is often important to distinguish between these two entities. It is also important to be aware of pseudo-lesions for accurate diagnosis. Inferior vena cava filter placement and endovascular treatment of the aorta are commonly performed procedures that can be associated with devastating complications, which are luckily infrequent. The calibre of the IVC also has its own clinical significance. Inferior vena cava pathologies, although rare, have a dramatic impact on the patient’s outcome and knowledge of these pathologies is prudent.

**Contribution:**

Understand the principles of IVC imaging, the common as well as the rare primary and secondary IVC tumours, differentiate between tumour thrombus and bland thrombus, and recognise IVC lesion mimics and life-threatening pathologies involving the IVC.

## Introduction

The inferior vena cava (IVC) is an uncommon site of pathology, although neoplastic and non-neoplastic pathologies can be recognised during abdominal imaging. Tumours of the IVC can be either primary or secondary, and they are often difficult to distinguish on imaging. Primary neoplasms of the IVC are uncommon. The IVC is more commonly secondarily involved in abdominal malignancies. Pertinent imaging findings help in differentiating between benign and malignant IVC thrombus. Pseudolesions need to be differentiated from true intraluminal filling defects to prevent misdiagnosis. Awareness of the normal imaging features of IVC stents and fistula, as well as normal postsurgical changes in transplant patients enable accurate detection of complications. We hope this article will increase the understanding of common as well as rare, acquired pathologies affecting the IVC.

## Imaging

Ultrasonography (US), along with colour Doppler imaging, is a cost effective, radiation free, easily available imaging modality, and is often used for the initial evaluation of IVC pathologies, especially in paediatric patients. However, it remains limited because of operator dependence and poor evaluation of the infrahepatic segment of the IVC by bowel gas obscuration. Nevertheless, Doppler US is often the initial imaging modality used for evaluation of flow in the IVC, site of occlusion or stenosis. Ultrasonography can also complement CT and MRI in evaluation of Budd-Chiari syndrome, depiction of the tumour thrombus and the differentiation of pseudo-thrombus from true thrombus.^1,2,3^

**FIGURE 1:**
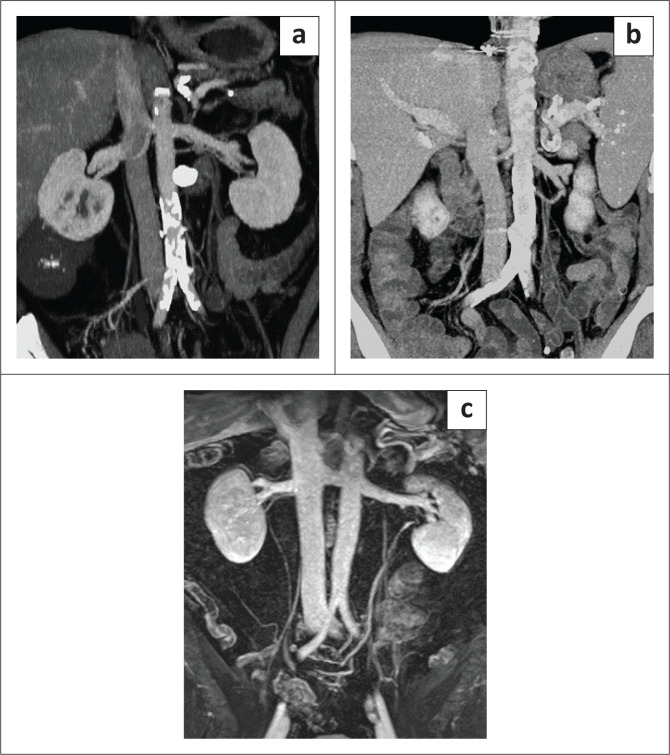
(a) Coronal CT abdomen in the portal venous phase showing denser contrast mixing into the inferior vena cava (IVC). (b) Coronal CT abdomen image acquired at 90 s showing homogeneous enhancement of the IVC. (c) Indirect contrast-enhanced magnetic resonance venography image demonstrating a normal IVC to the right of the aorta.

Post-contrast CT of the abdomen is the preferred and most commonly used modality for the evaluation of the IVC. The inferior vena cava is often evaluated on routine CT abdomen and pelvis, which is usually obtained in the post-contrast portal venous phase at 60 s – 70 s. However, in the portal venous phase, there is differential contrast enhancement of the renal and suprarenal IVC compared with the infrarenal portion. Venous return from the kidneys result in denser contrast in the renal and suprarenal IVC. Thus, portal venous phase imaging may result in incomplete opacification of the infrarenal IVC and may show admixture artifact. Increasing the delay time for CT image acquisition to 70 s – 90 s after intravenous contrast administration allows homogeneous opacification of the infrarenal IVC (Figure 1). With the advancement of multidetector computed tomography (MDCT) scanners, multiplanar reformation can be obtained, which is often useful in delineating IVC disease.^1,2,3,4^

The use of MRI not only avoids the risk of ionising radiation but also helps in a more reliable detection of the presence and delineation of tumour thrombus extent when compared with CT. However, anaesthesia for the paediatric population, cost and limited availability are challenges for the routine use of MRI for IVC assessment. Evaluation of the IVC can be performed using post-contrast three-dimensional breath-hold T1-weighted MR images. Balanced steady-state free precession is another useful MRI sequence.^2,3^ Magnetic resonance venography (MRV) is often used for evaluation of venous structures mostly in the brain but can also be used for evaluation of the IVC. It can be obtained without gadolinium, using time of flight (TOF) or phase techniques.^5,6^ The TOF sequence uses the flow related enhancement technique where stationary tissues are saturated and only protons flowing into the slice can generate signal. Application of a pre-saturation band to the artery proximal to the slice can avoid signal from inflowing arterial blood. The TOF sequence can be a 2D or 3D acquisition, but 3D TOF is rarely used for body MRV because of the long scan time. 2D TOF is prone to respiratory artifact and has poor resolution. In addition, if the vein imaged is oriented along the plane of imaging, there can be loss of signal because of saturation of inflowing protons.^5^ The phase contrast technique uses phase shift and velocity differences of moving protons for imaging. Sensitivity to a particular vessel is based on velocity encoding. Vessels with flow velocity close to velocity encoding are more sensitive. Contrast-enhanced MRV can be performed, and is superior in the evaluation of the IVC, as it depends on gadolinium rather than flow phenomena and hence is less prone to artifacts.^7^ It can be performed with an indirect or direct approach. For the indirect approach, contrast is injected in a peripheral vein with imaging performed during the early equilibrium phase (Figure 1). A large amount of contrast is required because of the dilution of contrast. This method is not preferred in abdominal MRV because of misregistration from respiratory motion. Using the direct approach, diluted contrast is continuously injected upstream of the venous territory. The amount of contrast required is considerably less because of targeted injection of dilute contrast, but results in good image resolution as a result of a superior signal-to-noise ratio.^5,8^ The 3D contrast-enhanced MRV techniques have a wide range of applications including evaluation of IVC abnormalities.^7^

## Inferior vena cava neoplasms

Inferior vena cava neoplasms are rarely primary and are more commonly secondary involvement from abdominal malignancy.^2^

### Inferior vena cava haemangioma

**FIGURE 2:**
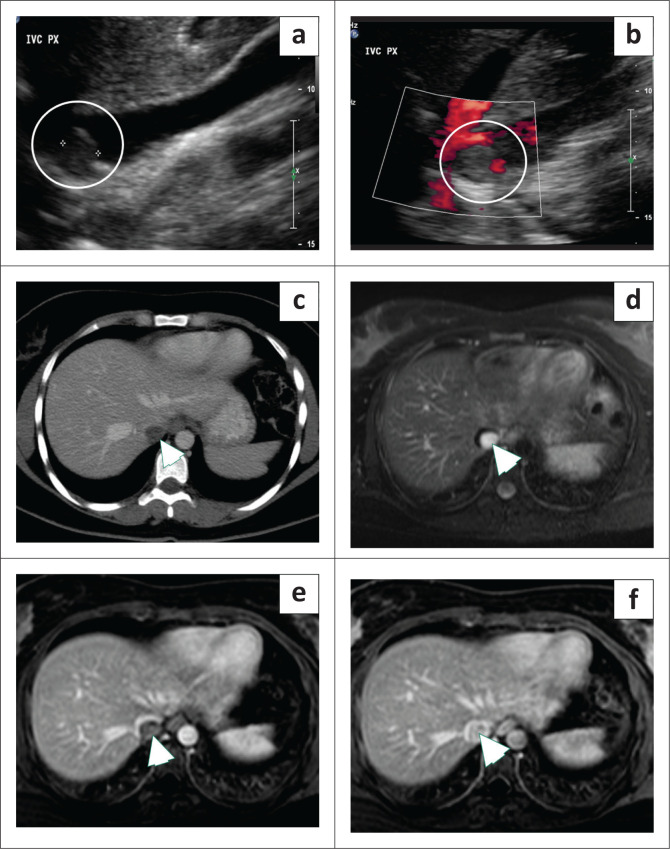
A 36-year-old female with acute abdominal pain related to cholelithiasis and an incidental inferior vena cava (IVC) finding on upper abdominal ultrasound. (a) Greyscale ultrasound image showed a 1.5 cm hypoechoic lesion (circle) within the IVC lumen. (b) The colour Doppler image shows no significant internal flow within the lesion (circle). (c) Contrast-enhanced CT of the abdomen in the portal venous phase did not reveal significant enhancement (arrowhead). (d) Axial T2-weighted images of the abdomen show hyperintense signal within the IVC (arrowhead). Dynamic contrast-enhanced MRI (e) portal venous phase and (f) delayed sequence acquired at 5 min after the injection demonstrate progressive enhancement with centripetal fill in suggesting the diagnosis of an IVC haemangioma (arrowhead).

Haemangiomas at most locations demonstrate characteristic features allowing for a confident radiological diagnosis. However, they can occasionally occur at atypical sites and have atypical imaging features that pose a diagnostic dilemma and may necessitate surgical excision. Inferior vena cava haemangiomas are rare with a few reported cases in the literature.^9,10^ Multiphase CT or MRI are often needed for the diagnosis demonstrating an intraluminal mass within the IVC, indistinguishable on imaging from other IVC neoplasms (Figure 2).^10^

### Primary inferior vena cava leiomyoma

**FIGURE 3:**
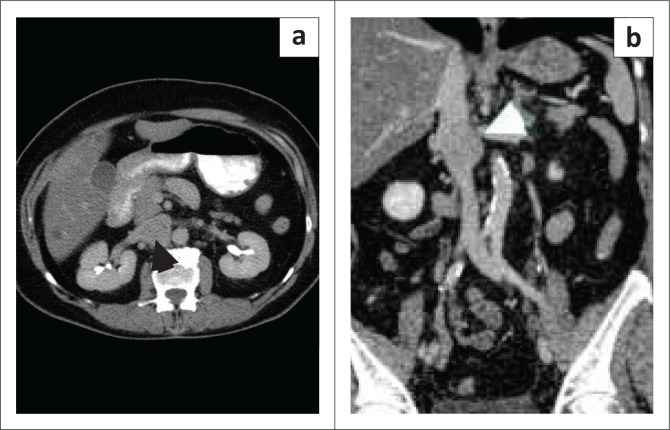
A 52-year-old female who was suspected to have a retroperitoneal mass during right upper quadrant ultrasound for abdominal pain. Contrast-enhanced axial CT (a) and coronal (b) showed a soft tissue mass (arrowhead) arising from the IVC. This proved to be an IVC leiomyoma on histopathology.

This is a benign tumour that may be seen in the IVC and is rarer than IVC leiomyosarcoma. It accounts for 15% of tumours arising from large veins.^11^ Imaging features are non-specific, demonstrating a solid mass within the IVC that is difficult to differentiate from other IVC tumours (Figure 3). These tumours, like their uterine counterpart, may appear heterogeneous after degeneration and demonstrate calcification, cystic changes, or haemorrhage. Extraluminal extension and invasion of the adjoining organs are important differentiating features of malignant involvement of the IVC from benign IVC leiomyoma.^2,3^

### Intravascular papillary endothelial hyperplasia

**FIGURE 4:**
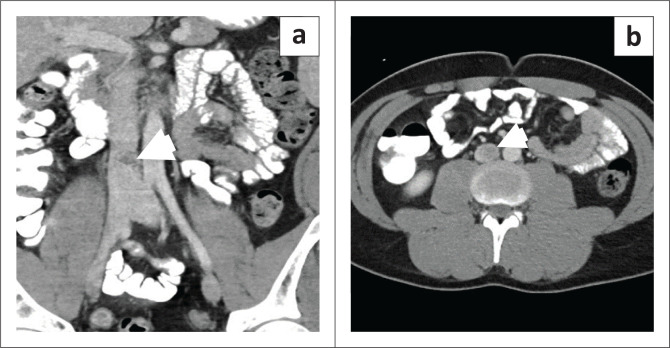
A 49-year-old male with non-specific abdominal pain was found to have a filling defect (arrowhead) in the inferior vena cava on the contrast-enhanced coronal CT image (a) and axial (b), which was histopathologically proven to be papillary endothelial hyperplasia.

Intravascular papillary endothelial hyperplasia (IPEH) is a benign, non-neoplastic entity of intravascular origin. It is also known as Masson’s tumour, intravascular angiomatosis, Masson’s pseudo-angiosarcoma, and vegetant intravascular haemangioendothelioma.^12^ It is a rare entity that results from excessive proliferation of endothelial cells, mediated by elevated fibroblast growth factor in an attempt to recanalise a thrombosed vein. It can occur in normal blood vessels or vascular malformations.^13^ Intravascular papillary endothelial hyperplasia can be classified into three subtypes. The most common subtype is the pure form which arises in dilated vessels. The mixed form arises in pre-existing vascular lesions such as haemangioma, aneurysm, vascular malformation and pyogenic granuloma. The third subtype is an extravascular form, which arises within haematomas.^14^ It predominantly involves the skin and subcutaneous tissues of the head and neck and extremities as well as the oral cavity.^15,16^ Rarely, large veins such as the renal vein, external jugular vein and superior vena cava can be involved.^14,17^ Intravascular growth of these entities have been reported to result in obstruction leading to raised intracranial pressure^18^ and superior vena cava syndrome.^19^ To the authors’ knowledge, involvement of the IVC has not been reported and the images of the patient presented likely represent the first case of IVC involvement by IPEH. Intravascular papillary endothelial hyperplasia can mimic aggressive vascular tumours such as endothelioma and angiosarcoma.^20^ Imaging features are not characteristic, appearing echogenic with significant vascularity on ultrasound and as homogeneous masses with intense enhancement on CT (Figure 4). On MRI, these lesions depict T1 intermediate and T2 heterogeneous hyperintense signal.^21^

### Primary inferior vena cava leiomyosarcoma

**FIGURE 5:**
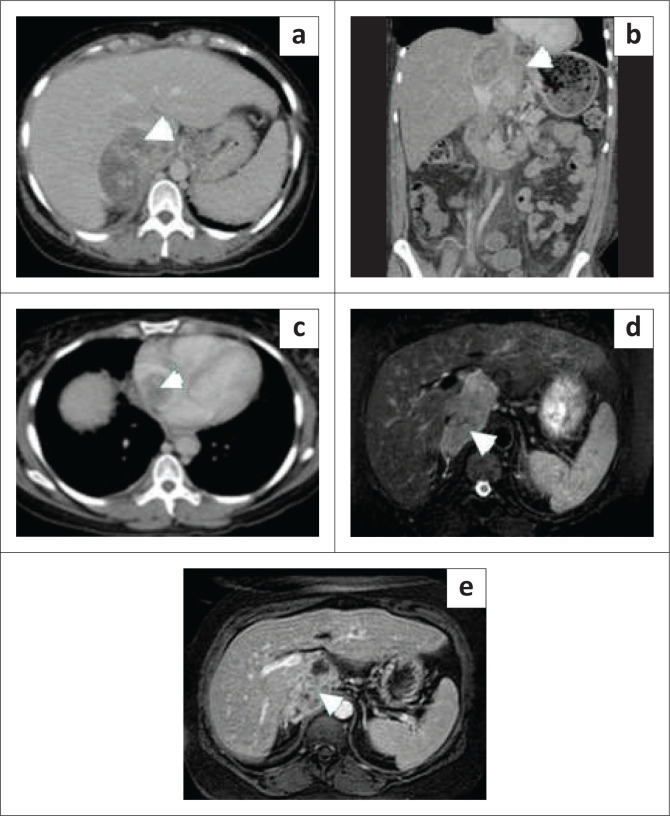
Inferior vena cava (IVC) leiomyosarcoma. (a) Contrast-enhanced axial CT abdomen showed a heterogeneous enhancing mass (arrowhead) expanding the IVC lumen. (b) The coronal contrast-enhanced CT image showed the mass (arrowhead) has a significant exophytic component in the retroperitoneum. (c) The axial CT image demonstrated extension of the mass (arrowhead) into the right atrium. (d) The axial T2-weighted fat saturated image showed a slightly T2 hyperintense mass (arrowhead) expanding the IVC lumen. (e) The post-contrast axial fat saturated image showed a heterogeneously enhancing mass (arrowhead) in the IVC lumen.

This is an uncommon malignancy, accounting for less than 1% of all malignancies. Yet, it is the most common primary malignancy involving the IVC.^22^ Nearly three-quarters of these tumours arising from large veins are leiomyosarcomas.^2^ These tumours originate from the smooth muscle cells of the vessel wall. Nearly three quarter of these cases occur in middle-aged women.^23^ Most leiomyosarcomas are large at presentation. They present with non-specific symptoms such as abdominal pain, a palpable mass and lower limb oedema. Inferior vena cava leiomyosarcoma initially has intramural growth, but ultimately two-thirds have extraluminal growth.^24,25^ Intraluminal growth causes venous obstruction while extraluminal growth results in invasion of adjacent structures. Involvement of the renal and suprarenal IVC is seen in nearly half of the patients, and is associated with a better prognosis, while intrahepatic IVC involvement seen in less than 20% of cases, is associated with a worse prognosis.^2^ On imaging, it appears as a mass filling the IVC with heterogeneous enhancement. Non-enhancing areas within the mass represent necrosis (Figure 5). En bloc resection of the IVC and grafting is often performed as complete surgical resection is necessary for cure. The overall outcome is poor with a 14% 10-year survival rate and 50% recurrence.^1,2^

### Intracaval extension of benign tumours

Benign tumours extending into the IVC are extremely rare. Benign leiomyomas of the uterus, although uncommon, may show intracaval extension. They may demonstrate intravenous growth via the pelvic veins with extension into the IVC and right atrium. The leiomyoma is continuous with the parent pelvic tumour and not fixed to the IVC wall, unlike primary IVC leiomyosarcoma. To prevent recurrence, complete transcaval excision along with total abdominal hysterectomy and bilateral salpingo-oophorectomy is necessary. Other benign tumours with reported intracaval extension include renal angiomyolipoma and adrenal pheochromocytoma. Unlike benign pheochromocytoma that extends into the adrenal vein, its malignant counterpart directly invades the IVC wall.^3,26^

### Inferior vena cava invasion and malignant thrombus

**FIGURE 6:**
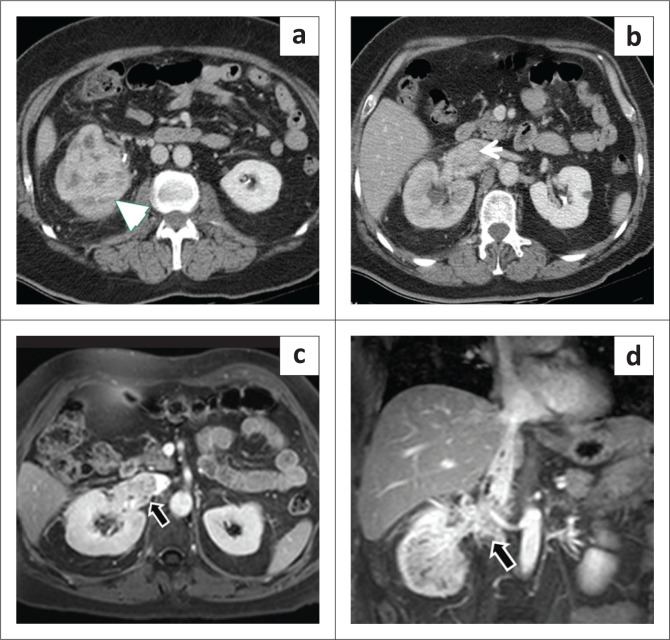
(a, b) The axial contrast-enhanced CT images demonstrated a heterogeneously enhancing mass (arrowhead) arising from the right kidney, which was proven to be renal cell carcinoma with an enhancing filling defect (thin white arrow) in the inferior vena cava. (c, d) The axial and coronal post-contrast T1-weighted fat saturated images revealed enhancement within the filling defect suggestive of tumour thrombus (black arrow).

**FIGURE 7:**
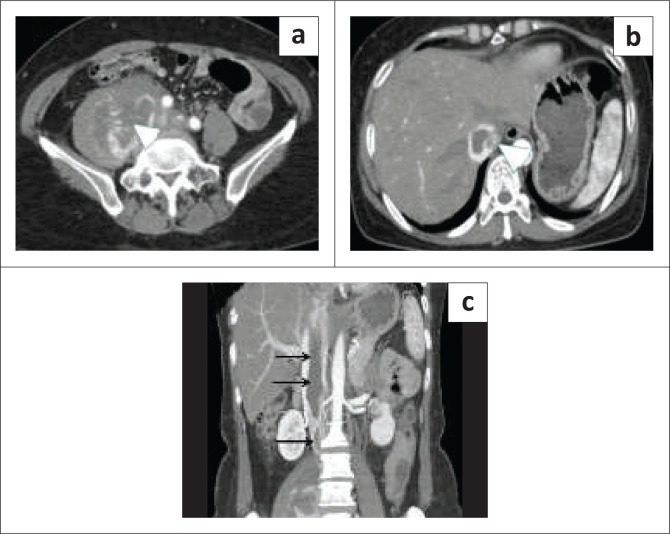
(a) Heterogeneous enhancing mass in the pelvic retroperitoneum (arrowhead) involving the psoas muscle, diagnosed as a sarcoma. (b) Intraluminal tumour thrombus extending up to the intrahepatic inferior vena cava (IVC) (arrowhead). In contrast to the bland thrombus, the tumour thrombus component shows enhancement. (c) The coronal CT shows the extent of IVC thrombus (black arrows).

Malignant involvement of the IVC may be because of direct invasion of the IVC wall or endovascular extension with and without intraluminal thromboembolisation. Tumour thrombus is the most common manifestation of malignant involvement of the IVC, which can be differentiated from bland thrombus on the basis of expansion of the vessel lumen, enhancement of the filling defect and direct continuity between the primary tumour and the thrombus^27,28^ (Figure 6 and Figure 7). Correct identification of the extent of IVC involvement is essential for appropriate staging and surgical intervention.^2^ Renal cell carcinoma (RCC), hepatocellular carcinoma (HCC), adrenocortical carcinoma and Wilms tumour commonly show direct contiguous tumour extension into the IVC.^28^ Renal cell carcinoma in particular shows little tendency to invade the venous wall. If tumour thrombus is seen infiltrating the venous wall, segmental resection of the IVC becomes essential, but involvement of the venous wall is difficult to assess with imaging alone and is usually diagnosed only at surgical exploration. Malignant thrombus in the IVC is clinically asymptomatic most of the time but can occasionally result in Budd-Chiari syndrome or embolise into the pulmonary circulation.^3^

Renal cell carcinoma is the most common malignancy extending into the IVC. Inferior vena cava involvement is seen in 4% – 10% of patients with RCC. Imaging with CT is the first choice for RCC as it not only accurately identifies IVC extension of the RCC but allows for a simultaneous metastatic survey. Further subclassification into infrahepatic, hepatic, and suprahepatic extension of the tumour thrombus is critical to predict the complexity of the resection and the surgical approach. In the T3c stage, the infradiaphragmatic portion of the IVC is involved and extension into the supradiaphragmatic IVC advances the tumour to the T4b stage. Supradiaphragmatic extension requires cardiopulmonary bypass during the surgical procedure, increasing morbidity and mortality during the procedure. Sagittal and coronal reconstructions are useful to reliably delineate the extent of tumour thrombus and differentiate it from bland thrombus by depicting direct continuity. Arterial recruitment from adjacent organs suggests vessel wall invasion.^1,2^

Transitional cell carcinoma may result in a filling defect in the IVC and renal vein. Extension into the IVC is rare but invasion of the IVC wall is more common in transitional cell cancer than in RCC. The patient may require segmental IVC resection and the prognosis is poor.^2^

Wilms tumour is the most common renal tumour in children and is associated with IVC invasion in 4% – 8% of cases.^29^ Although US can show vascular extension, CT and MR imaging are better for evaluation of metastatic disease. Recognition of IVC involvement is important because it upstages the tumour from I to II and stage II tumour may necessitate neoadjuvant chemotherapy or radiation therapy. Inferior vena cava extension increases morbidity during nephrectomy.^2^

Adrenal cortical carcinoma is a rare malignancy. It is seen as a heterogeneous mass replacing the entire adrenal gland, frequently seen with calcification on imaging. Patients typically present with an advanced stage and there is a high risk of recurrence. Intravascular extension into the IVC may be seen in nearly one-third of the cases. It occurs more commonly in right-sided tumours and tumours measuring greater than 9 cm.^2,30^

While HCC typically results in portal venous thrombosis, 4.0% – 5.9% of patients can have hepatic venous and IVC invasion. They can also extend into the right atrium and result in Budd-Chiari syndrome.^11,27^ Systemic venous invasion and extension into the right atrium is associated with extremely poor prognosis and a high risk of distant metastasis.

Non-seminomatous testicular tumours can also involve the IVC with tumour thrombus from intravascular spread through gonadal veins or direct invasion of IVC wall by metastatic retroperitoneal lymphadenopathy. Bulky retroperitoneal lymph nodes and IVC tumour thrombus in a young patient should prompt further evaluation with US of the scrotum for testicular malignancy.^2^

## Bland thrombus

**FIGURE 8:**
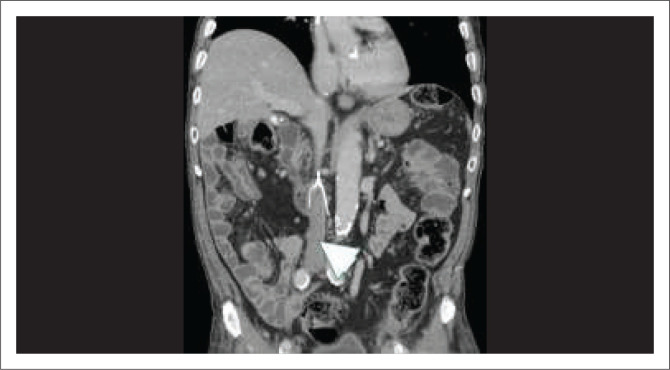
The coronal contrast-enhanced CT image of the abdomen showed an inferior vena cava (IVC) filter in place with a non-enhancing intraluminal filling defect (arrowhead) in the IVC extending all the way to the level of the IVC filter.

Bland thrombus in the IVC usually occurs because of spread of thrombus from the lower extremity veins but can be isolated. It predisposes to pulmonary embolism. Causes include hypercoagulable states and malignancies as well as local factors such as compression of the IVC by fibrosis, lymph nodes or retroperitoneal masses. Sluggish venous flow and the presence of foreign bodies such as IVC filters or catheters also increase clot formation (Figure 8). Bland thrombus results in a persistent filling defect in the vessel on post-contrast studies. Collateral veins form around the IVC and aorta in chronic extensive thrombosis to bypass the obstruction. Absence of luminal expansion and enhancement favours the diagnosis of bland thrombus over tumour thrombus (Figure 7). Anticoagulation is the mainstay of therapy.

Placement of an IVC filter is an alternative option for patients who cannot be anticoagulated. Gonadal vein thrombophlebitis in postpartum patients can also result in bland thrombus that may extend into the IVC. It can cause expansion and enhancement of the venous wall because of perivascular inflammation and mimic tumour thrombus. It is treated with anticoagulation and antibiotics.^1^ Endovascular treatment such as catheter-directed thrombolysis and angioplasty can rapidly restore flow in patients with acute on chronic bland thrombus.^26^

## Pseudolesions

Artifactual filling defects can be seen when unopacified blood from the lower extremity without contrast mixes with blood from the renal veins with dense contrast. It can be misinterpreted as clot by an inexperienced reviewer. Another potential mixing artifact can be seen with laminar reflux of contrast mixed blood into the hepatic veins from the heart. This is often seen in right heart failure or with a rapid contrast injection rate faster than 3 mL/s and can also mimic clot. Delayed imaging can be performed at 90 s for problem solving in equivocal cases with complete opacification of the IVC in pseudolesions.

**FIGURE 9:**
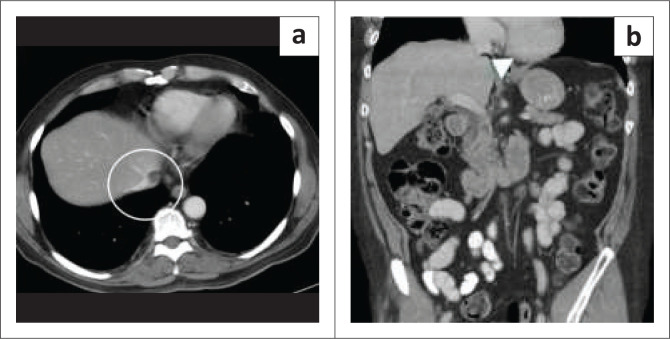
A 47-year-old male evaluated for abdominal pain. The axial (a) and coronal (b) contrast-enhanced CT images demonstrated juxtacaval fat (arrowhead) mimicking an inferior vena cava tumour or thrombus (circle).

Cirrhotic patients often have prominent fat around the IVC above the caudate lobe. Tilting of the IVC because of right hepatic lobe atrophy may give the appearance of fat projecting into the lumen of the IVC, mimicking a lipoma, hence called pseudolipoma (Figure 9).^1,2,3^

## Thrombosed aneurysm of the inferior vena cava

Inferior vena cava aneurysms are rare. Aneurysms likely arise because of a congenital weakness of the wall. The normal diameter of the infrahepatic IVC in the adult ranges from 1.5 cm to 3.7 cm on CT. Membranous obstruction of the IVC is a congenital anomaly which has been associated with aneurysm formation. Inferior vena cava aneurysms may be saccular, fusiform or diverticular.^31,32^ Symptomatic aneurysms (expansion, rupture, or embolisation) will probably require surgical management.^31^

## Budd-Chiari syndrome

Budd-Chiari syndrome results from obstruction of the hepatic vein outflow tract. Budd-Chiari syndrome is most commonly associated with a hypercoagulable state. Uncommonly congenital web, stenosis or tumour can result in obstruction of the hepatic segment of the IVC. Inferior vena cava compression and narrowing by a massively enlarged caudate lobe is another uncommon cause.^1^ The caudate lobe undergoes hypertrophy in most of the cases and is relatively spared of the effects of Budd-Chiari as it drains directly into the IVC. However, caudate lobe hypertrophy is a non-specific finding. On the other hand, a prominent caudate lobe vein measuring more than 2 mm is a specific sign seen in half of the patients with Budd-Chiari syndrome.^3^

**FIGURE 10:**
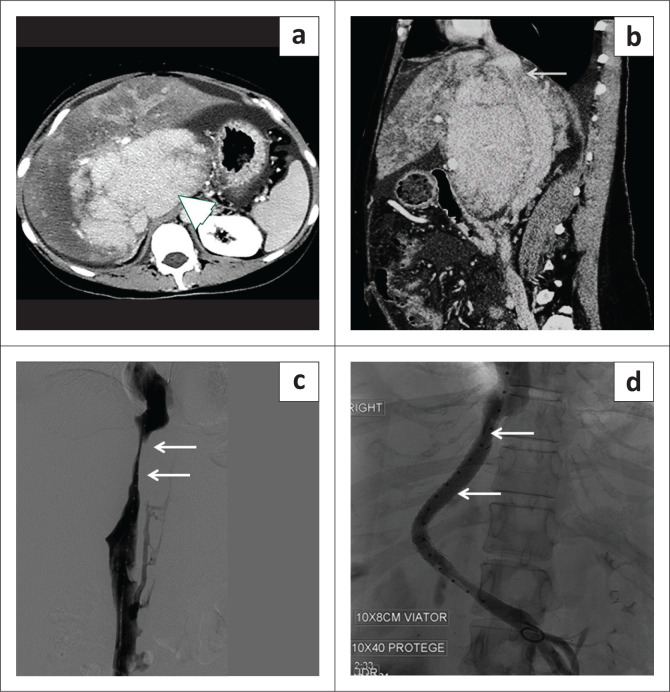
(a) Contrast-enhanced CT demonstrates preferential enhancement of the central liver of liver (white arrowhead) in a patient with Budd-Chiari syndrome. (b) Sagittal CT abdomen showing narrowing of the infrahepatic inferior vena cava (white arrowhead) and an enlarged caudate. (c) Venogram confirms the diagnosis. (d) Venogram placement of porto-systemic venous shunt.

In the acute phase, arterial phase imaging demonstrates preferential enhancement of the central region of the liver, which includes the hypertrophied caudate lobe and part of the left lobe with washout of contrast in the central region and accumulation of contrast peripherally in the portal venous phase (flip-flop pattern of enhancement) (Figure 10).^3^

In chronic Budd-Chiari syndrome, the imaging appearance depends on the contrast phase and regenerative nodules. Regenerative nodules may be seen as hyperattenuating lesions with hypervascular enhancement in the arterial phase but most are difficult to identify on imaging because of their small size. On MRI, regenerative nodules have variable signal on T1-weighted images, are iso-hypointense on T2-weighted images, and do not enhance in the arterial phase. On the contrary, HCC shows heterogeneous enhancement on the arterial phase, are encapsulated and invade the portal and hepatic veins.^1,3^ Benign nodules are hyperintense to normal liver on post-contrast delayed imaging with hepatocyte-specific phase agents such as gadobenate compared with HCC.^3^ Azygos, hemiazygos, and lumbar veins bypass the occluded IVC segment via collaterals.^1^ Extrahepatic collaterals may be seen in Budd-Chiari as well as other IVC syndromes because of hepatic outflow obstruction but the presence of intrahepatic collaterals is highly suggestive of Budd-Chiari.^30^

## Inferior vena cava stents and filters

Inferior vena cava stents are often used to bypass occluded or narrowed segments of the IVC. Inferior vena cava stenosis often occurs after longstanding indwelling venous catheters or at anastomotic sites in liver transplant patients. Multidetector CT is very useful for the evaluation of the stent integrity and to confirm its patency.

**FIGURE 11:**
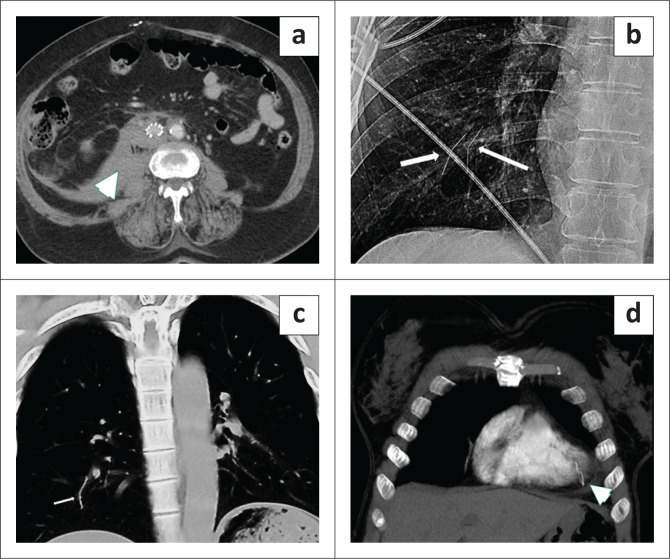
(a) Axial CT abdomen demonstrating a retroperitoneal haematoma (arrowhead) from perforation of the inferior vena cava (IVC) by the IVC filter. (b, c) Chest X-ray and coronal CT thorax showing migrated IVC filter struts (white thin arrows) in the right lower lobe pulmonary artery branches. (d) Embolism of the IVC filter strut into the right ventricle (arrowhead).

Inferior vena cava filter placement is a safe option for patients with deep vein thrombosis who cannot be anticoagulated. Imaging with MDCT can assess the location of IVC filters and identify potential complications such as thrombosis, migration, embedding in the IVC wall as well as fracture and embolism of fracture fragments.^1^ Perforation of the IVC by the filter strut is commonly seen and are usually asymptomatic but cases of acute retroperitoneal haematoma because of perforation of the IVC have been reported^33,34^ (Figure 11a). Retroperitoneal haematoma from injury to the lumbar artery after IVC filter placement has also been reported.^35,36^ Fracture of the IVC filter struts with migration to the heart, pulmonary vasculature and renal veins have been reported with some requiring surgical intervention^37^ (Figure 11b–d). Other complications include perforation of the duodenum, aorta and renal pelvis after IVC filter placement.^37^

## Inferior vena cava fistulas

**FIGURE 12:**
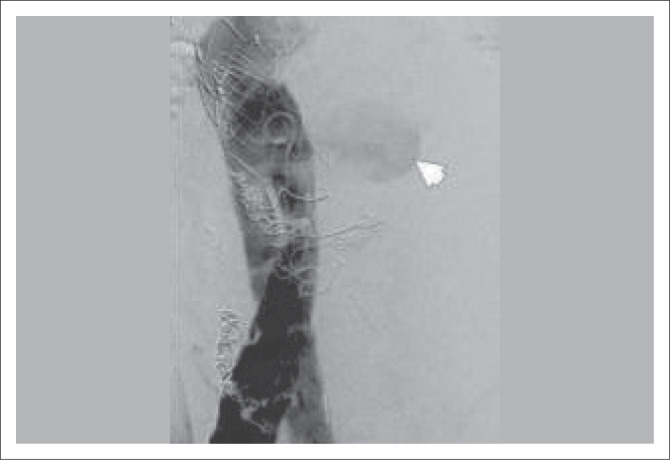
Intraoperative angiogram performed during endovascular aneurysm repair stent placement demonstrates inferior vena cava opacification (arrowhead) suggestive of an iatrogenic aortocaval fistula formation.

The presence of an arteriovenous fistula or a significant arteriovenous malformation is suggested on imaging by enhancement of the IVC in the arterial phase. Rarely fistulas can form between the IVC and adjacent organs which can be life threatening. Similarly, aortocaval fistula is rare (prevalence 0.2% – 0.9%) (Figure 12). Most patients present with acute symptoms of high output congestive heart failure. More than 80% of cases result from an abdominal aortic aneurysm rupturing or eroding into the IVC.^2^ The remaining cases occur secondary to trauma. Rarely, neoplastic and inflammatory aetiologies result in formation of a fistula. In addition to early contrast opacification of the IVC in the arterial phase, loss of the normal fat plane between the aorta and IVC, and increase in calibre of the IVC from high volume flow are important findings. Prompt recognition and early treatment are crucial for improved patient outcomes.^1,3^

## Flattened or slit-like inferior vena cava

**FIGURE 13:**
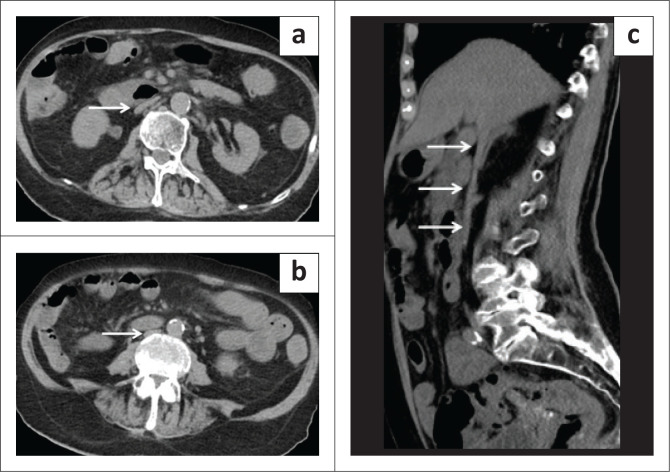
An 83-year-old female with gastrointestinal bleeding, haemorrhagic shock and renal dysfunction. Unfortunately, the patient expired within a few hours of imaging. The CT axial and sagittal images show a markedly flattened inferior vena cava (white thin arrows).

A slit-like IVC is defined as the transverse IVC diameter greater than three times the anteroposterior diameter of the IVC when measured at multiple levels.^1,3^ A flattened IVC can be seen in trauma patients with marked hypotension (Figure 13) and impending shock. However, this is a non-specific finding and nearly two-thirds of the patients with this imaging finding can be euvolemic and normotensive, particularly in the non-traumatic scenario and in elderly women in whom it can be a normal variant.^1^ Additional useful signs that would favour hypovolaemia include a peripheral hypodense halo, small calibre of the abdominal aorta, increased enhancement of the bowel wall, kidneys, and pancreas and marked diffuse bowel distension. The presence of haemoperitoneum may suggest a flattened IVC to be a significant finding in a trauma patient.^1,3^

## Megacava

**FIGURE 14:**
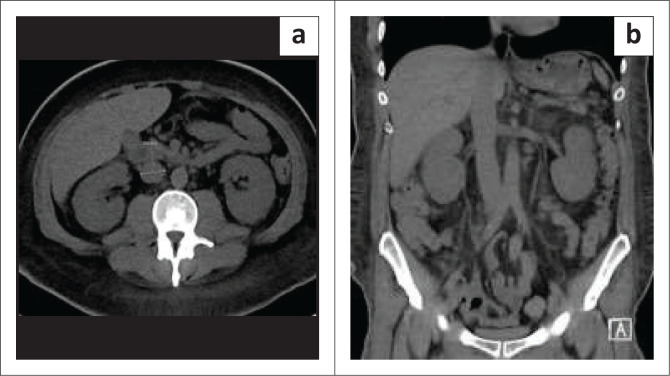
A 62-year-old with abdominal pain. Axial and coronal CT abdomen showing a prominent inferior vena cava measuring 29 mm in the infrarenal segment with anasarca probably from congestive heart failure.

A megacava has a prevalence of about 1% – 3%.^26,38^ They are frequently seen in patients with right heart failure.^26^ In early angiographic studies, the mean IVC diameter ranged between 20 mm and 23 mm in most patients. An infrarenal IVC greater than 28 mm in diameter is defined as megacava (Figure 14). This cutoff is used as an IVC greater than 28 mm size is associated with an higher chance of device failure and migration of IVC filters.^26,38^ Inferior vena cava filters designed for use in megacava measuring up to 40 mm are available but there is no retrievable IVC filter approved for use in megacava.^26,39^ The alternative option for patients with megacava is placement of filters in both iliac veins, which can be technically challenging because of iliac vein tortuosity.^40^

## Postsurgical changes

A mesocaval shunt is surgically created by establishing a connection between the superior mesenteric vein and the IVC to decrease portal pressure. Although it has fallen out of favour with the advent of the transjugular intrahepatic portosystemic shunt (TIPS) procedure, it may still be useful for decompression of variceal bleeding when patients have portal vein occlusion and a TIPS procedure is technically difficult or impossible. These shunts can now be created using an endovascular technique and intravascular US guidance. A mesocaval shunt may be uncommonly encountered incidentally or for the assessment of patency.^2^

Liver transplantation frequently utilises postoperative imaging for evaluation of treatment complications. Graft failure in liver transplant patients is most common because of acute rejection followed by vascular complications. Postoperative swelling can cause compression and stenosis of the IVC after liver transplantation. Interrogation of the stenotic site using spectral Doppler demonstrates three- to four-fold increase in velocity without phasicity, normally seen in the hepatic veins because of atrial modulation.^2^

## Conclusion

There is a broad spectrum of pathologies affecting the IVC including neoplastic and non-neoplastic aetiologies Reporting the extent of thrombus and differentiating between bland versus malignant thrombus has a direct bearing on patient care. Awareness of the normal imaging appearances of IVC stents and fistulas along with normal postoperative changes enables accurate detection of complications. It is important to know the pitfalls in IVC imaging to differentiate between true filling defects and pseudolesions.
